# Supplementary material for: Interfacial polarization of in vivo rat sciatic nerve with crush injury studied via broadband dielectric spectroscopy
Source: PLoS One. 2021 Jun 2;16(6):e0252589. doi: 10.1371/journal.pone.0252589 (PMC8171940; doi:10.1371/journal.pone.0252589)
Supplement: S1 Table — (DOCX) [file pone.0252589.s001.docx]

S1 Table. Samples informations.

| Sample  ID number | Time | Rat | Mesurement site | Voltage V | Distance between IC clips | Diameter of nerve *Φ* | Color in Figs. 3–9 |
| --- | --- | --- | --- | --- | --- | --- | --- |
|  |  |  |  | [V] | [mm] | [mm] |  |
| 1 | 11:55 | A | Non-injury | 1.00 | No record | No record | ― |
| 2 | 11:02 | A | Non-injury | 1.00 | 8.7 | No record | ― |
| 3 | 11:05 | A | Non-injury | 1.00 | 4.9 | No record | ― |
| 4 | 11:32 | A | Injury site | 0.100 | 11.6 | 1.8 | ― |
| 5 | 11:35 | A | Injury site | 1.00 | 11.6 | 1.8 | ― |
| 6 | 11:38 | A | Injury site | 0.500 | 11.6 | 1.8 | ― |
| 7 | 11:59 | B | Non-injury | 0.500 | 6.6 | 1.3 | ― |
| 8 | 12:02 | B | Non-injury | 0.100 | 6.6 | 1.3 | ― |
| 9 | 12:20 | B | Injury site | 0.500 | No record | 1.7 | ― |
| 10 | No record | B | Injury site | 0.100 | No record | 1.7 | ― |
| 11 | 12:26 | B | Proximal site | 0.100 | 5.5 | 1.7 | ― |
| 12 | No record | C | Non-injury | 0.100 | 5.8 | 1.0 | ― |
| 13 | No record | C | Non-injury | 0.500 | 5.8 | 1.0 | ― |
| 14 | 14:41 | C | Injury site | 0.100 | 8.3 | 0.9 | ― |
| 15 | 14:45 | C | Injury site | 0.500 | 8.3 | 0.9 | ― |
| 16 | 14:54 | C | Proximal site | 0.500 | 5.4 | 0.9 | ― |
| 17 | 15:05 | D | Non-injury | 0.100 | 6 | 1.0 | ― |
| 18 | No record | D | Non-injury | 0.500 | 6 | 1.0 | ― |
| 19 | 15:23 | D | Injury site | 0.100 | 7.5 | 1.4 | ― |
| 20 | 15:26 | D | Injury site | 0.500 | 7.5 | 1.4 | ― |
| 21 | 15:30 | D | Proximal site | 0.500 | 6.8 | 1.4 | ― |
| 22 | 15:33 | D | Proximal site | 0.500 | 6.8 | 1.4 | ― |
| 23 | 15:43 | E | Non-injury | 0.100 | 5.4 | 1.2 | ― |
| 24 | 15:47 | E | Non-injury | 0.500 | 5.4 | 1.2 | ― |
| 25 | 15:58 | E | Injury site | 0.100 | 6.6 | 1.6 | ― |
| 26 | 16:02 | E | Injury site | 0.500 | 6.6 | 1.6 | ― |
| 27 | 16:11 | E | Proximal site | 0.500 | 4.1 | 1.6 | ― |
| 28 | 16:15 | E | Distal site | 0.500 | 5.5 | 1.6 | ― |
| 29 | 17:12 | F | Non-injury | 0.100 | 7.4 | 1.4 | ― |
| 30 | 17:16 | F | Non-injury | 0.500 | 7.4 | 1.4 | ― |
| 31 | 17:42 | F | Injury site | 0.100 | 7.2 | 0.9 | ― |
| 32 | 17:45 | F | Injury site | 0.500 | 7.2 | 0.9 | ― |
| 33 | 17:49 | F | Proximal site | 0.500 | 4.1 | 0.9 | ― |
| 34 | 17:56 | F | Distal site | 0.500 | 4.2 | 0.9 | ― |
