# Supplementary material for: Interfacial polarization of in vivo rat sciatic nerve with crush injury studied via broadband dielectric spectroscopy
Source: PLoS One. 2021 Jun 2;16(6):e0252589. doi: 10.1371/journal.pone.0252589 (PMC8171940; doi:10.1371/journal.pone.0252589)
Supplement: Attachment — Submitted filename: PONE-D-21-05156.docx [file pone.0252589.s002.docx]

The submission from Rita Otagiri et al. reports that the strength of injured nerve interfacial polarization was smaller than that observed in nerves without an injury. Moreover, authors suggest that BDS could be used as a tool to understand the electrical aspect of the pathophysiology of nerve injury. The study is interesting and well structured. However, there are some corrections.

***Minor comments:***

1. ***The authors should add some more current references about sciatic nerve with crush injury (***doi: [10.3390/ijms21103509](https://dx.doi.org/10.3390%2Fijms21103509)***;*** doi: [10.1186/s12974-018-1303-5](https://dx.doi.org/10.1186%2Fs12974-018-1303-5))
2. ***The authors should include in the materials and methods section the experimental groups they analyzed during the experiment.***
3. ***The authors should better check the manuscript for any typographical errors.***
