# Supplementary material for: Interfacial polarization of in vivo rat sciatic nerve with crush injury studied via broadband dielectric spectroscopy
Source: PLoS One. 2021 Jun 2;16(6):e0252589. doi: 10.1371/journal.pone.0252589 (PMC8171940; doi:10.1371/journal.pone.0252589)
Supplement: Attachment — Submitted filename: Respond_Letter.docx [file pone.0252589.s003.docx]

Response to Reviewers

We sincerely thank the reviewers for their thoughtful comments, which helped us improve our manuscript. The manuscript has been revised to address the reviewers’ concerns. Revised Pages mentioned below are from the revised manuscript with red words.

[Comment 1 ]

The authors should add some more current references about sciatic nerve with crush injury *(*doi: [10.3390/ijms21103509](https://dx.doi.org/10.3390%2Fijms21103509)*;* doi: [10.1186/s12974-018-1303-5](https://dx.doi.org/10.1186%2Fs12974-018-1303-5))

[Response 1 ]

Thank you for the suggestion. We have cited the two references (#19 and 20) regarding sciatic nerve with crush injury, as suggested by you.

[Comment 2 ]

The authors should include in the materials and methods section the experimental groups they analyzed during the experiment.

[Response 2 ]

Thank you for the suggestion. As suggested, we have added a sentence describing the experimental groups analyzed in the study under the subsection titled “Animals.”

[Comment 3]

The authors should better check the manuscript for any typographical errors.

[Response 3]

We apologize for the typographical errors. The manuscript has been carefully proofread for language and spellings by a professional English language editing company.
